# Comorbidities and Environmental Factors Associated with Atopic Dermatitis in Children and Adults in Dermatology-Venereology in Cotonou, Benin

**DOI:** 10.1155/2021/1502721

**Published:** 2021-11-24

**Authors:** Bérénice Dégboé, Félix Atadokpèdé, Christabelle Nguessie, Alida Kouassi, Nadège Elégbédé, Nina Maffo, Fabrice Akpadjan, Hugues Adégbidi

**Affiliations:** Department of Dermatology Venereology, National Teaching Hospital Hubert Koutoukou Maga of Cotonou, Faculty of Health Sciences, University of Abomey-Calavi, Godomey, Benin

## Abstract

**Introduction:**

The objective of this work was to document the comorbidities and environmental factors associated with atopic dermatitis (AD) in dermatology Venereology in Cotonou.

**Methods:**

A cross-sectional, prospective, and analytical study included, from January 2016 to December 2018, in the Dermatology-Venereology Department of the National Teaching Hospital Hubert Koutoukou Maga (CNHU-HKM) of Cotonou, children and adults after free and informed consent, in whom the diagnosis of AD was retained according to the criteria of the United Kingdom Working Party. Severity was assessed using SCORAD (severity scoring of atopic dermatitis).

**Results:**

The overall prevalence of AD was 7.7%. AD was more frequent in children (56.8% and 40.6%) and adults (59.8% and 37.4%) from urban and periurban areas (0.003 < *p* < 0.034). It was more frequent in children who regularly dewormed and those with complete vaccination (0.001 < *p* < 0.01). In 54.8% of children and 58.9% of adults, flare-ups occurred during the warm season. The main associated comorbidities were rhinitis and conjunctivitis in both children (49.7% and 36.1%, respectively) and adults (32.7% and 26.2%, respectively). The main triggering factors in children were heat (43.2%), pneumallergens (28.4%), and skin irritants (22.6%). In adults, we noted skin irritants (58.9%), heat (47.7%), and psychological factors (34.6%). In adults, the use of detergent soaps was associated with lichenified and severe AD (0.003 < *p* < 0.006) and that of lightening soaps with acute AD (*p*=0.042).

**Conclusion:**

AD in the Dermatology-Venereology Department of the CNHU-HKM of Cotonou was associated with comorbidities. It was influenced by environmental factors related to the tropical climate and by skin irritants or allergens.

## 1. Introduction

Atopic dermatitis (AD) is a chronic and recurrent pruritic inflammatory dermatosis that preferentially affects infants. Worldwide, its prevalence has doubled or even tripled in the last 30 years [1–3].

Several studies show that it is not a simple disease, but a skin condition with many different genetic and immunological mechanisms and underlying environmental factors. These factors influence the prevalence and clinical expression of the disease in different age groups, geographical regions, and races [4–8].

In sub-Saharan Africa and particularly in Benin, very few studies have been carried out in this field. It therefore seems appropriate to document the environmental factors and comorbidities associated with AD in children and adults in the Dermatology-Venereology Department of the National Teaching Hospital “Hubert Koutoukou Maga” (CNHU-HKM) of Cotonou.

## 2. Patients and Methods

A cross-sectional, prospective, descriptive, and analytical study was carried out in the Dermatology-Venereology Department of the CNHU-HKM in Cotonou. The National Teaching Hospital CNHU-HKM of Cotonou is a public healthcare. Cotonou is the economic capital of Benin characterized by high air pollution and a high population. This region is under the influence of a tropical climate with a long dry season from December to March, followed by a long rainy season from April to July, and then a short dry season from August to September and a short rainy season from October to November. Temperatures vary between 28 and 32° during the dry season and between 23 and 26° during the rainy season. Wind speed during the year varies from 9 to 16.6 km per hour. Throughout the year, there is a very high humidity above 79%.

The study included, from January 2016 to December 2018, children and adults diagnosed with AD, after the free and informed consent of adult patients and regarding to children, those of their parents. The study was approved by the department head. The diagnostic criteria of the United Kingdom Working Party were used for the diagnosis of AD in children under 10 years of age [1]. In children over 10 years of age and adults, anamnestic (personal history of pruritic dermatosis, personal or family history of asthma, and/or allergic rhinitis or other atopic manifestations) and clinical (morphological and topographical aspects characteristic of AD in older children, adolescents, and adults, the presence of minor signs of atopy) arguments were used to establish the diagnosis of AD [1, 9, 10]. Sampling was exhaustive, nonprobabilistic, and nonrandomized and included all patients admitted to dermatology consultations who met the inclusion criteria.

According to the period of onset in adults, AD was classified as persistent when the first onset occurred in childhood, before 18 years of age, and late when the first onset occurred after this period [9]. The degree of severity was assessed with the SCORAD (scoring of atopic dermatitis).

The triggering and/or aggravating environmental factors were identified on the basis of the patients' allegations. The triggering factors sought were pneumallergens (house dust mites, pollen, mold, pet dander, and smoke), infections, teething in infants, skin irritants (perfumed products, detergents, and synthetic or woolen clothing), trophallergens (milk, egg white, peanuts, soy, shellfish, and okra), psychological factors (stress and psychoaffective conflicts in adolescents and adults), hormonal factors in girls (premenstrual period), and physicochemical and climatic factors (sweat, heat, cold, and harmattan). Allergological tests, such as specific immunoglobulin E assays, prick tests, and patch tests, were not available to assess the relevance of the environmental factors reported by the patients. We relied on a thorough medical history (police-like questioning) to determine the notion of the cause and effect in the occurrence or recurrence of AD flare-ups in these patients. In case of a negative or doubtful answer, no factor was retained.

The study was approved by local ethics committee of faculty of health sciences of Cotonou.

Demographic, socioeconomic, and clinical data were entered and analyzed with Epi-Data 3.1 and Epi-Info 7.0 softwares. The search for links between the occurrence of dermatosis and the other independent variables under study was carried out by univariate analysis; Pearson's chi-square or Fischer's test was used to search for the association between the variables. Results were significant when *p* < 0.05.

## 3. Results

In the Dermatology-Venereology Department of the CNHU-HKM in Cotonou, over the study period, we received 958 children and 2434 adults. Of these 958 children and 2434 adults, 155 children and 107 adults had AD. The prevalence of AD in the pediatric population was 16.2% and 4.4% in adults. This gives an overall hospital prevalence of 7.7%. The M/F sex ratio was 0.8 in both populations.

As given in Table 1, children between 0 and 5 years of age made up the majority of patients, 51.6% of the pediatric population, while young adults between 19 and 30 years of age made up the majority (41.1%) of the adult population. The median age at the time of the first episode was 4.6 years ± 4.5 in children and 69.7% of them; 108 children had their first episode before the age of 5 years. Among adults, the median age at the time of the first episode was 31.6 years ± 17, and 86% had a late onset of AD versus 14% who had a persistent AD.

The main associated comorbidities were rhinitis and conjunctivitis in both children and adults but in different proportions: 49.7% of cases of rhinitis and 36.1% of cases of conjunctivitis in children against 32.7%, and 26.2%, respectively, in adults. Asthma was present in 16.1% of children (25 children) and was more frequent in adolescents between 16 and 18 years of age who suffered from AD (*p*=0.001). These results are given in Table 2.

AD was more common in patients who had more than one other associated allergic disease (*p*=0.001). AD was more common in children who regularly dewormed (*p*=0.01) and those with complete vaccination (*p*=0.001). Almost all children (151; 97.4%) and adults (104; 97.2%) were from urban and periurban areas and 0.003 < *p* < 0.034. In 54.8% of children (85) and 58.9% of adults (63), the flare-ups occurred during the warm season. The main triggering factors in children were heat (67; 43.2%), pneumallergens (44; 28.4%), and skin irritants (35; 22.6%). In adults we noted skin irritants (63; 58.9%), heat (51; 47.7%), and psychological factors including stress (37; 34.6%). The illustration of these results is given in Table 2.

The average SCORAD was 30.6 ± 15.1 in children and 30.2 ± 12.9 in adults. In adults, the use of detergent soaps was associated with lichenified and severe AD (0.003 < *p* < 0.006) and that of lightening soaps with acute AD (*p*=0.042).

## 4. Discussion

The lack of valid criteria for the diagnosis of AD in older children over 10 years of age, adolescents, and adults constitutes limitations to our study. However, the anamnestic and clinical criteria used, combined with the experience of the dermatologists in the department, can significantly reduce the margins of diagnostic error. Another limitation is the failure to perform allergological tests to confirm the relevance of the triggering and/or aggravating factors reported by the patients. This is mainly due to the unavailability of these tests in Benin. Nevertheless, we estimated, given the paucity of studies on AD in sub-Saharan Africa, to include patients according to this anamnestic criterion and compare with previous studies' results. We hope that this work, although imperfect, can form a preliminary basis for more advanced studies in the future.

The prevalence of AD in the Dermatology-Venereology Department of CNHU-HKM in Cotonou in 2009 was 5.5% [11]. It rose to 7.7% in 2018. There is therefore a 40% increase in the hospital prevalence of AD over 10 years. This prevalence is slightly lower than that obtained in Nigeria, which was 8.5% in 2000 [12]. Our study confirms the high prevalence of AD in children (16.2%). This pediatric prevalence is significantly higher than that reported in Abidjan (Côte d'Ivoire) in 2017 [13]. Several studies confirm the increase in AD prevalence over the last 10 years, particularly in developing countries [4–6]. In Africa, the prevalence of AD varies from country to country between 4.7% and 23% [14]. Genetic factors alone are therefore not sufficient to explain the increase in AD around the world. There is a complex interrelation between these factors and environmental factors, which partly explains this observed disparity.

The comorbidities frequently found in both children and adults were rhinitis and conjunctivitis. According to some authors, AD is considered the first manifestation of atopic gait, followed by food allergy, asthma, and rhinitis or rhinoconjunctivitis [2, 13, 15]. These allergic manifestations are often found in high proportions in atopic patients. [12, 13, 16–18]. According to some authors, the risk of developing allergic rhinitis and asthma in the presence of AD is more or less important [1, 2, 16, 19].

Considered as the key initiating event of atopic march, the alteration of the skin barrier accounts for the link between atopic dermatitis and subsequent atopic diseases [14, 19–22]. In our study, asthma, which is significantly more frequent in adolescents, and the multiplicity of allergic diseases, associated with the onset of AD, confirm this hypothesis.

However, the notion of an atopic march has recently been controversial. On the one hand, some authors believe that allergic manifestations, collected on the basis of patient claims, are overestimated in most studies [23, 24]. On the other hand, AD is not always associated with other allergic manifestations. It has been suggested that these different manifestations, while sharing genetic and environmental risk factors, are independent conditions that can develop concomitantly or sequentially on an atopic site [16, 24]. However, the concept of atopic march offers the possibility of research on the pathogenesis prospects for the prevention and treatment of atopic diseases. Correct measures to maintain or restore skin barrier function may help minimize the risk of developing allergic manifestations.

AD in Cotonou was more frequent in cases of regular deworming against helminthiasis or full vaccination, especially in children. The results of studies concerning hygiene theory are contradictory. Some studies have reported that there is no link between helminthiasis and the onset of AD. Other studies have shown that helminthiasis is a protective factor against allergic diseases including AD [8, 25–27]. According to the authors of the hygiene theory, the presence of helminthiasis induces the secretion of anti-inflammatory cytokines and/or an increased response of suppressor T lymphocytes. The reduction of infectious diseases through vaccination and deworming leads to a reorientation of the immune system towards the Th2 mechanism and so an increased sensitization to allergens [15, 19, 25–29]. The hygiene theory also supports the fact that the lack of stimulation of the body by microbes is a risk factor for the onset of atopic diseases. Recent studies show conflicting results: protection from the measles vaccine and worsening by other vaccines, especially when these are given early. The short and long-term responses of viruses and bacteria to immune responses and DA expression remain to be elucidated [8, 26, 30–33].

In univariate analysis, residence in urban and periurban areas is significantly associated with the occurrence of AD. A systematic review of the literature conducted by Uphoff et al. in 2015 [34] showed that in most cases, children living in highly industrialized environments, with a Western lifestyle and a high socioeconomic level, have a higher risk of developing allergy. Urbanization, improvement of hygiene and food quality, reduction of microbial infections, vaccination, use of antiinfective, and increased exposure to pneumallergens are factors implicated in the higher prevalence of atopic diseases in urban areas [8, 14, 29, 35–37]. Associated with this lifestyle, we can also mention air pollution, which is a determining environmental factor. Atmospheric pollution leads to an imbalance of oxidative stress at the level of the skin barrier [36, 38]. The synergistic effect of all of these factors could contribute to the onset and/or worsening of AD. A better knowledge of these factors will make it possible to act with those which can be modified.

Heat was a reported contributing factor for both children and adults. The majority of flare-ups occurred during the hot season. The main climatic factors reported in Nigeria, a country bordering ours, are heat, excessive sweating, and humidity [12]. A high temperature causes sweating, which becomes more important when humidity increases. This weather condition is the one observed in our region, hence the high frequency of this factor. Sweat can irritate the skin due to its acidic pH. This could promote Th2 inflammation, increased skin blood flow, and a pruritogenic mechanism via nerve endings in the skin. There is not only a correlation between temperature and other climatic factors, namely, humidity, exposure to ultraviolet rays but also the pollen concentration in the environment, alteration of barrier function, and skin irritants. This indicates the important role of climate and even climate change, in the increase in flare-ups of AD [4, 8, 36, 38].

Our study also confirms the role of skin irritants in the occurrence of flare-ups. Intrinsic barrier dysfunction can be aggravated when environmental factors such as soap and detergents cause further degradation of the epidermal barrier and irritants and allergens can interact with the immune system and promote inflammation. These irritants are thought to promote the synthesis of immunoglobulin E and sensitization to allergens [38]. Similarly, the use of detergent or lightening soaps was associated with specific clinical forms of AD.

## 5. Conclusion

In the Dermatology-Venereology Department of CNHU-HKM of Cotonou, atopic dermatitis was associated with other atopic manifestations, the most frequent of which were rhinitis, conjunctivitis, and asthma. Environmental factors such as regular deworming, full vaccination, and living in an urban area were associated with the occurrence of AD. Heat associated with high humidity, skin irritants, and pneumallergens were reported in significant proportions.

Multivariate analysis studies on a large series in the general population, supported by the demonstration of these factors using allergological tests, and will provide a better understanding of these factors and their role in the onset or aggravation of atopic dermatitis in our regions. All these will contribute to a better knowledge of the pathophysiology of the disease and to a better therapeutic and above all preventive approach.

## Figures and Tables

**Table 1:** Age distribution of 155 children and 107 adults with AD in the Dermatology Department of the CNHU-HKM in Cotonou from January 2016 to December 2018.

		Number	Proportion (%)
Children	0–5	80	51.6
	6–10	41	26.5
	11–15	24	15.5
	16–18	10	6.4
	Total	**155**	**100**

Adults	19–30	44	41.1
	31–40	28	26.2
	41–50	16	14.9
	51–60	11	10.3
	>60	8	7.5
	Total	**107**	**100**

**Table 2:** Triggering or aggravating environmental factors in 155 children and 107 adults with AD in the Dermatology Department of the CNHU-HKM in Cotonou from January 2016 to December 2018.

		Children		Adults	
		Number (%)	*P* value	Number (%)	*P* value
Comorbidities	Rhinitis	**77 (49.7)**	0.756	**35 (32.7)**	0.656
	Conjunctivitis	**56 (36.1)**	0.402	**28 (26.2)**	0.408
	Asthma	**25 (16.1)**	**0.001**	16 (15)	0.276
	Sinusitis	5 (3.2)	0.353	27 (25.2)	0.780
	Food allergy	4 (2.6)	—	4 (3.7)	—
	Drug allergy	0 (0)	—	3 (2.7)	—
	Prurigo strophulus	5 (3.2)	—	0 (0)	—

Antecedents	Deworming	**92 (59.4)**	**0.011**	50 46.7	0.732
	Vaccination	**85 (54.8)**	**0.001**	—	—

Residence	Urban	**88 (56.8)**	**0.003**	**64 (59.8)**	**0.034**
	Periurban	**63 (40.6)**		**40 (37.4)**	
	Rural	4 (2.6)		3 (2.8)	

Period of last outbreak	Dry season	**85 (54.8)**	0.489	**63 (58.9)**	0.61
	Rainy season	70 (45.2)		44 (41.1)	

Triggering and/or aggravating factors	Heat	**67 (43.2)**	—	**51 (47.7)**	—
	Pneumallergens	**44 (28.4)**	—	**37 (34.6)**	—
	Skin irritants	**35 (22.6)**	—	**63 (58.9)**	—
	Trophallergens	**29 (18.7)**	—	14 (13.1)	—
	Psychological	23 (14.8)	—	**38 (33.6)**	—
	Infections	15 (9.7)	—	—	—
	Teething	11 (7.1)	—	—	—

The important values are highlighted in bold.

## Data Availability

The data related to this study and results can be consulted in the archives in the Dermatology Department of CNHU-HKM of Cotonou and are available from the corresponding author upon request.

## References

[1] Taïeb A. (2005). Dermatite atopique : définition, épidémiologie, histoire naturelle, gravité et scores. *Annales de Dermatologie et de Venereologie*.

[2] Mahé E. (2005). Dermatite atopique: épidémiologie en France, définitions, histoire naturelle, association aux autres manifestations atopiques, scores de gravité, qualité́ de vie. *Annales de Dermatologie et de Venereologie*.

[3] Ezzedine K., Kechichian E. (2017). Épidémiologie de la dermatite atopique. *Annales de Dermatologie et de Vénéréologie*.

[4] Schmid‐Grendelmeier P., Takaoka R., Ahogo K. C. (2019). Position statement on atopic dermatitis in sub‐saharan Africa: current status and roadmap. *Journal of the European Academy of Dermatology and Venereology*.

[5] Torrelo A. (2014). Atopic dermatitis in different skin types. What is to know?. *Journal of the European Academy of Dermatology and Venereology*.

[6] Kim Y., Blomberg M., Rifas-Shiman S. L., Camargo C. A., Gold D. R., Thyssen J. P. (2018). Racial/Ethnic differences in incidence and persistence of childhood atopic dermatitis. *Journal of Investigative Dermatology*.

[7] Zar H. J., Ehrlich R. I., Workman L., Weinberg E. G. (2007). The changing prevalence of asthma, allergic rhinitis and atopic eczema in African adolescents from 1995 to 2002. *Pediatric Allergy & Immunology*.

[8] Bonamonte D., Filoni A., Vestita M., Romita P., Foti C., Angelini G. (2019). The role of the environmental risk factors in the pathogenesis and clinical outcome of atopic dermatitis. *BioMed Research International*.

[9] Reguiaï Z. (2017). ’Dermatite atopique de l’adulte : présentation clinique, complications et comorbidités. *Annales de Dermatologie et de Vénéréologie*.

[10] Silvestre Salvador J., Romero-Pérez D., Encabo-Durán B. (2017). Atopic dermatitis in adults: a diagnostic challenge. *Journal of Investigational Allergology and Clinical Immunology*.

[11] Atadokpèdé́ F., Adégbidi H., Koudoukpo C., Agbessi N., Dégboé́-Sounhin B., Akpadjan F. (2012). Dermatite atopique au Benin: aspects cliniques et thérapeutiques. *Dakar Medical*.

[12] Nnoruka E. N. (2004). Current epidemiology of atopic dermatitis in south-eastern Nigeria. *International Journal of Dermatology*.

[13] Ahogo K. C., Kouassi Y. I., Gbery I. P., Azagoh K. R., Yeboua K. I., Kouassi K. A. (2017). Atopic dermatitis in children: epidemiological and clinical aspects in Côte d’Ivoire. *Our Dermatology Online*.

[14] Kaufman B. P., Guttman-Yassky E., Alexis A. F. (2018). Atopic dermatitis in diverse racial and ethnic groups-Variations in epidemiology, genetics, clinical presentation and treatment. *Experimental Dermatology*.

[15] Chiesa Fuxench Z. C. (2017). Atopic dermatitis: disease background and risk factors. *Advances in Experimental Medicine and Biology*.

[16] Ćosićkić A., Skokić F., Selimović A., Mulić M., Suljendić S., Nermina Dedić N. (2017). Development of respiratory allergies, asthma and allergic rhinits in children with atopic dermatitis. *Acta Clinica Croatica*.

[17] Chu H., Shin J. U., Park C. O., Lee H., Lee J., Lee K. H. (2017 March). Clinical diversity of atopic dermatitis: a review of 5,000 patients at a single institute. *Allergy, Asthma & Immunology Research*.

[18] Técléssou J. N., Mouhari-Toure A., Akakpo S., Bayaki S., Boukari O. B. T., Elégbédé Y. M. (2016). Facteurs de risque et manifestations allergiques associés à la dermatite atopique à Lomé (Togo): étude multicentrique portant sur 476 enfants de 0 à 15 ans. *Medecine et Sante Tropicales*.

[19] Taniuchi S., Soejima K., Hatano Y., Takahashi M., Minami H. (2018). Dual factors may Be necessary for development of atopic march in early infancy. *Journal of Nippon Medical School*.

[20] van den Oord R. A. H. M., Sheikh A. (2009). Filaggrin gene defects and risk of developing allergic sensitization and allergic disorders: systematic review and meta-analysis. *BMJ*.

[21] Čepelak I., Dodig S., Pavić I. (2019). Filaggrin and atopic march. *Biochemical Medicine*.

[22] Dharmage S. C., Lowe A. J., Matheson M. C., Burgess J. A., Allen K. J., Abramson M. J. (2014). Atopic dermatitis and the atopic march revisited. *European Annals of Allergy and Clinical Immunology*.

[23] Busse W. W. (2018). The atopic march: fact or folklore?. *Annals of Allergy, Asthma, & Immunology*.

[24] Yang L., Fu J., Zhou Y. (2020). Research progress in atopic march. *Frontiers in Immunology*.

[25] Heratizadeh A., Werfel T., Kapp A. (2006). Atopic dermatitis: the hygiene hypothesis: prevention through helminth infections?. *Hautarzt, Der*.

[26] Flohr C., Yeo L. (2011). Atopic dermatitis and the hygiene hypothesis revisited. *Current Problems In Dermatology*.

[27] Staal S. L., Hogendoorn S. K. L., Voets S. A., Tepper R. C., Veenstra M. (2018). Prevalence of atopy following mass drug administration with albendazole: a study in school children on flores island, Indonesia. *Allergy and Immunology*.

[28] Smits H. H., Everts B., Hartgers F. C., Yazdanbakhsh M. (2010). Chronic helminth infections protect against allergic diseases by active regulatory processes. *Current Allergy and Asthma Reports*.

[29] Collège des Enseignants de Pneumologie (2017). *Référentiel ECN : Item 182 : Hypersensibilités et allergies respiratoires chez l’adulte : aspects physiopathologiques, épidémiologiques, diagnostiques et principes de traitement*.

[30] Hennino A., Cornu C., Rozieres A., Augey F., Villard-Truc F., Payot F. (2007). Influence of measles vaccination on the progression of atopic dermatitis in infants. *Pediatric Allergy & Immunology*.

[31] Gehrt L., Rieckmann A., Kiraly N., Jensen A. K. G., Aaby P., Stabell Benn C. S. (2020). Timeliness of DTaP-IPV-hib vaccination and development of atopic dermatitis between 4 Months and 1 Year of age-register-based cohort study. *Journal of Allergy and Clinical Immunology*.

[32] Adler U. C. (2005). The influence of childhood infections and vaccination on the development of atopy: a systematic review of the direct epidemiological evidence. *Homeopathy*.

[33] Randi G., Altieri A., Chatenoud L., Chiaffarino F., La Vecchia C. (2004). Infections and atopy: an exploratory study for a meta-analysis of the hygiene hypothesis. *Revue d’Epidemiologie et de Sante Publique*.

[34] Uphoff E., Cabieses B., Pinart M., Valdés M., Antó J. M., Wright J. (2015). A systematic review of socioeconomic position in relation to asthma and allergic diseases. *European Respiratory Journal*.

[35] Kathuria P., Silverberg J. I. (2016). Association of pollution and climate with atopic eczema in US children. *Pediatric Allergy & Immunology*.

[36] Nguyen G. H., Kronborg Andersen L., Davis M. D. P. (2018). Climate change and atopic dermatitis: is there a link?. *International Journal of Dermatology*.

[37] Morgenstern V., Zutavern A., Cyrys J., Brockow I., Koletzko S., Krämer U. (2008). Atopic diseases, allergic sensitization, and exposure to traffic-related air pollution in children. *American Journal of Respiratory and Critical Care Medicine*.

[38] Kantor R., Silverberg J. I. (2017). Environmental risk factors and their role in the management of atopic dermatitis. *Expert Review of Clinical Immunology*.

